# Highly Efficient Ultra-Thin EML Blue PHOLEDs with an External Light-Extraction Diffuser

**DOI:** 10.3390/nano13162357

**Published:** 2023-08-17

**Authors:** Shin-Woo Kang, Eun-Jeong Bae, Young-Wook Park, Byeong-Kwon Ju

**Affiliations:** 1 Display and Nanosensor Laboratory, Department of Electrical Engineering, Korea University, 145, Anam-ro, Seongbuk-gu, Seoul 02841, Republic of Korea; newoosw@korea.ac.kr (S.-W.K.);; 2 Nano and Organic-Electronics Laboratory, Department of Display and Semiconductor Engineering, Sun Moon University, Asan 31460, Republic of Korea

**Keywords:** organic light-emitting diodes (OLEDs), ultra-thin EML, undoped, phosphorescent, FIrpic, blue OLEDs, diffuser, light extraction, viewing angle, hemisphere, sphere, embedded

## Abstract

In this study, various diffusers are applied to highly efficient ultra-thin emission layer (EML) structure-based blue phosphorescent organic light-emitting diodes (PHOLEDs) to improve the electroluminescence (EL) characteristics and viewing angle. To achieve highly efficient blue PHOLEDs, the EL characteristics of ultra-thin EML PHOLEDs with the various diffusers having different structures of pattern–shape (hemisphere/sphere), size (4~75 μm), distribution (surface/embedded), and packing (close-packed/random) were systematically analyzed. The diffusers showed different enhancements in the overall EL characteristics of efficiencies, viewing angle, and others. The EL characteristics showed apparent dependency on their structure. The external quantum efficiency (EQE) was enhanced mainly by following the orders of pattern, size, and shape. Following the pattern size, the EQE enhancement gradually increased; the largest-sized diffuser with a 75 μm closed-packed hemisphere (diffuser-1) showed a 1.47-fold EQE improvement, which was the highest. Meanwhile, the diffuser with a ~7 μm random embedded sphere with a low density (diffuser 5) showed the lowest 1.02-fold-improved EQE. The reference device with ultra-thin EML structure-based blue PHOLEDs showed a maximum EQE of 16.6%, and the device with diffuser 1 achieved a maximum EQE of 24.3% with a 5.1% wider viewing angle compared to the reference device without a diffuser. For the in-depth analysis, the viewing angle profile of the ultra-thin EML PHOLED device and fluorescent green OLEDs were compared. As a result, the efficiency enhancement characteristics of the diffusers show a difference in the viewing angle profile. Finally, the application of the diffuser successfully demonstrated that the EL efficiency and viewing angle could be selectively improved. Additionally, we found that it was possible to realize a wide viewing angle and achieve considerable EQE enhancement by further investigations using high-density and large-sized embedded structures of light-extraction film.

## 1. Introduction

Phosphorescent organic light-emitting diodes (PHOLEDs), which are the most used among commercial OLEDs at present, have highly efficient characteristics [1,2,3,4]. Fluorescent OLEDs are characterized by using only singlet excitons for light-emission purposes; however, phosphorescent OLEDs utilize both singlet and triplet excitons for light-emission purposes. Theoretically, the maximum achievable internal quantum efficiency of PHOLEDs can be achieved at 100% [1,3]. However, due to the limitation of light out-coupling, the external quantum efficiency (EQE) is limited to about 20%. This is mainly due to optical confinement. Optical confinement mainly occurs due to the total internal reflection (TIR) in the multilayer stack of OLEDs with deferent refractive indices, and results from the surface plasmon polariton (SPP) mode at the organic/metal reflective electrode interface. Thus, approximately 80% of the light is trapped in the glass substrate as the substrate mode, in the indium tin oxide (ITO) anode and organic layers as the waveguide mode, and in the organic/metal electrode interface as the SPP mode [5,6]. The light-extraction technology used to achieve high-efficiency OLEDs is one of the main issues of OLED research. A micro-lens array (MLA) is a representative light-extraction technology that is effective for light-extraction practices without changing the internal structure of the device. The MLA avoids the total reflection of incident light due to the lens structure and increases the angular range at which light can be extracted. An OLED device with an MLA attached to it can extract waveguide- and substrate-mode lights while reducing the color coordinate change according to the viewing angle [7]. Another major research topic for OLEDs is the high efficiency results achieved through device physics [8,9]. One of the most widely used methods to increase OLED efficiency is the doping system. The doping system is mainly used in the emission layer (EML) of an OLED, and it is manufactured by co-depositing the doping layer host and dopant at a specific ratio. This method is the most traditionally used. However, by using the ultra-thin EML method, which forms a dopant thin film with a very low thickness (thinner 1 nm) between the hole transport layer (HTL) and electron transport layer (ETL), a similar effect to the doping system can be achieved without using the conventional doping method [10,11,12,13]. According to our group’s previous report, ultra-thin EML green PHOLEDs achieved a high EQE of about 24% [13].

In this study, ultra-thin EML-based blue PHOLEDs are fabricated. Additionally, various diffusers with different optical properties, such as transmittances and thicknesses, are applied to ultra-thin EML blue PHOLEDs to achieve high efficiency. The reference device achieved a maximum EQE of 16.6% and the device with a hemisphere-type diffuser showed the greatest enhancement with a maximum EQE of 24.3%.

## 2. Materials and Methods

### 2.1. Materials

Dipyrazino[2,3-f:2′,3′-h]quinoxaline-2,3,6,7,10,11-hexacarbonitrile (HAT-CN) was used as the hole injection layer, 4,4-Cyclohexylidenebis[N,N-bis(4-methylphenyl)benzenamine] (TAPC) was used as the HTL, Tris(4-carbazoyl-9-ylphenyl)amine (TCTA) was used as the HTL, Bis[2-(4,6-difluorophenyl)pyridinato-C^2^,N](picolinato)iridium (FIrpic) was used as the ultra-thin EML, 1,3,5-Tris(3-pyridyl-3-phenyl)benzene (TmPyPB) was used as the ETL, lithium fluoride (LiF) was used as the electron injection layer, and aluminum (Al) was used as the cathode. All organic materials were purchased from Sigma-Aldrich and Lumtec Corp. Diffusion layer 1 was purchased from MN Tech Co., Ltd., Cheongju-si, Republic of Korea, and diffusion layers 2~5 were purchased from Sun Green, Incheon, Republic of Korea.

### 2.2. Device Fabrication

**Figure 1:**
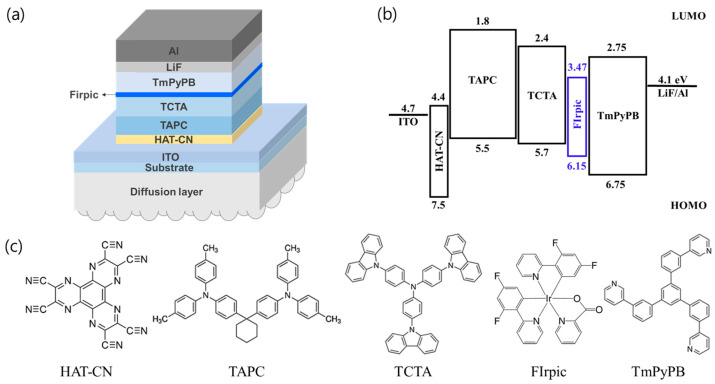
(**a**) Schematic of ultra-thin EML OLED device structure with diffuser. (**b**) Energy-level diagram of blue PHOLED device with ultra-thin EML structure. (**c**) Molecular structures of organic materials.

The OLEDs were fabricated on a commercially purchased ITO-coated glass substrate. The substrates were sequentially pre-cleaned via ultrasonication using acetone, methanol, and deionized (DI) water (Shinhan Science Tech, Daejeon, Republic of Korea). The active area of the OLED was defined by a 6.25 mm diameter circle using a photoresist (AZ 601 GXR, AZ Electronic Materials Co., Ltd., Darmstadt, Germany) via photolithography. Before thermal evaporation was conducted, the surface of the ITO was sequentially treated using ultraviolet (UV) ozone (UVC-300, Gyeonggi-do, Omniscience, Republic of Korea) and O_2_ plasma (CUTE, Femto Science Co., Hwaseong-si, Gyeonggi-do, Republic of Korea). The structure of the OLEDs was as follows: ITO/HAT-CN/TAPC/TCTA/FIrpic/TmPyPB/LiF/Al. The vacuum level of the process chamber was maintained at approximately 3 × 10^−7^ Torr. During the evaporation of all materials, the substrates were rotated at a constant speed of 12 rpm. The organic materials evaporated at a rate of approximately 1 Å/s, and the emitting dopant evaporated at an approximate rate of 0.01 Å/s. LiF and Al evaporated at a rate of approximately 0.1 Å/s and 4 Å/s, respectively. After the fabrication of ultra-thin EML blue PHOLEDs, various diffusers were attached to the substrate of the device. Figure 1 shows the schematic of the ultra-thin EML OLED structure with diffuser, band diagram of ultra-thin EML blue PHOLEDs, and the molecular structure of organic materials.

### 2.3. Measurement

We determined the thickness of the film during the high-vacuum thermal deposition of each thin film using a 6 MHz gold-coated quartz crystal microbalance (QCM, Phillip Technologies, Greenville, SC, USA) and thin-film deposition controller with a PCI Express interface (IQM-223, INFICON, Bad Ragaz, Switzerland). The thickness of the organic materials was measured using a QCM with lifetime value over 98%. The fabricated devices were stored in a glove box under an argon (Ar) atmosphere with less than 1 ppm of H_2_O. Using a thick film with thicker than 500 nm and a field-emission scanning electron microscope (SEM) (JSM-6700F, JEOL Co., Ltd., Tokyo, Japan) aided by a surface profilometer (Alpha-Step 500, KLA-Tencor, Milpitas, CA, USA), the thickness of each film was calibrated to precisely control the thickness of the ultra-thin films. The electroluminescence (EL) characteristics of the fabricated OLEDs were measured using a spectroradiometer (CS-2000A, Konica Minolta Co., Ltd., Tokyo, Japan) and source meter (Keithley 2400, Tektronix, Beaverton, OR, USA) in a dark box at 25 °C under low-vacuum conditions (~10^−3^ Torr) using a vacuum sample holder. The EQE was calculated using the viewing angle characteristics compared to the Lambertian light source. The viewing angle improvement was calculated by the intensity profile of the device according to the measurement angle (0~70°). The optical properties of the diffusers measured the total transmittance and perpendicular transmittance using a UV-Vis and near-infrared (NIR) spectrophotometer with an integrating sphere (Cary 5000, Agilent Technologies, Inc., Santa Clara, CA, USA) (Supplementary Materials Figure S1).

## 3. Results and Discussion

### 3.1. Determining the Ultra-Thin EML Thickness of Blue PHOLEDs

**Figure 2:**
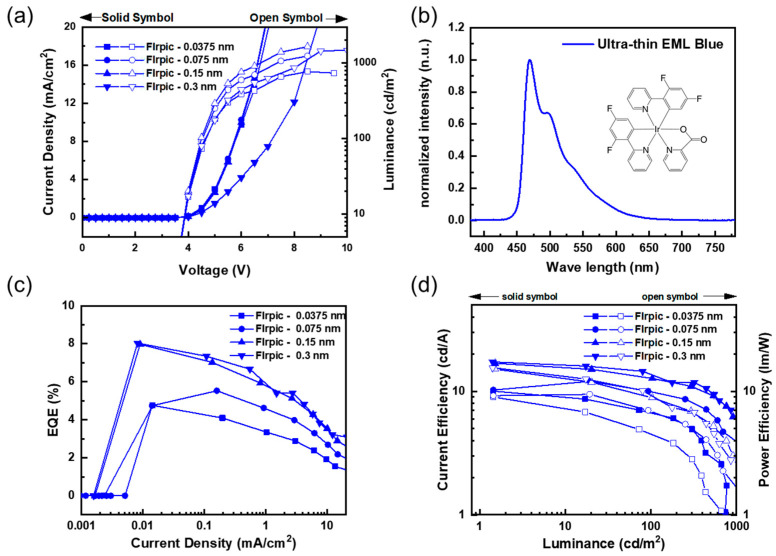
EL characteristics of ultra-thin EML blue PHOLEDs with various FIrpic thicknesses: (**a**) current density–voltage–luminance (*J–V–L*) characteristics, (**b**) EL spectra of ultra-thin EML blue PHOLEDs, (**c**) external quantum efficiency–current density (EQE–*J*) characteristics, and (**d**) current efficiency–luminance–power efficiency (*CE–L–PE*) characteristics.

In order to achieve the high efficiency of the ultra-thin EML blue PHOLEDs, the thickness values of ultra-thin EML, HTL, and ETL were precisely controlled. First, the ultra-thin EML thickness was adjusted and the device structure was used as follows: ITO (185 nm)/HAT-CN (1 nm)/TAPC (80 nm)/TCTA (30 nm)/FIrpic (X nm: 0.0375 to 0.3 nm)/TmPyPB (54 nm)/LiF (1.25 nm)/Al (200 nm). Figure 2 shows the EL characteristics according to the various EML thicknesses.

Figure 2a shows the current density–voltage–luminance characteristics. According to the thickness of the dopant, which exceeds 0.15 nm in ultra-thin EML blue PHOLEDs, the driving voltage tends to increase at the same current density. The increase in the driving voltage occurred because the dopant molecules acted as traps, and this could be interpreted as the driving voltage increasing because the hopping site increased. The maximum emission peak of the device with FIrpic can be observed at 470 nm in Figure 2b. Figure 2c,d shows the current density–EQE and current efficiency–luminance–power efficiency characteristics. The result shows that the thicker the dopant, the greater the EL efficiency. This tendency can be observed in the EQE, current efficiency, and power efficiency characteristics. However, it appears to be saturated at a thickness of 0.15 nm, and the thickness of 0.3 nm limits the increase in efficiency because energy transfer does not occur smoothly due to the trap site.

### 3.2. Optimization of Highly Efficient Blue PHOLEDs with Ultra-Thin EML Structure

**Figure 3:**
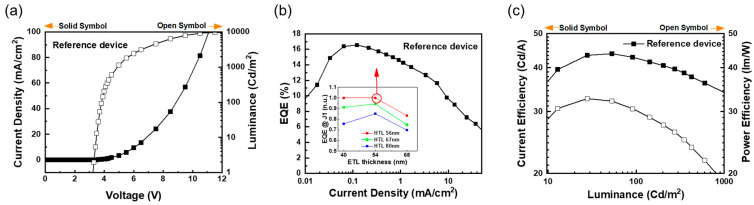
EL characteristics of optimized reference device: (**a**) Current density–voltage–luminance (*J–V–L*) characteristics; (**b**) external quantum efficiency–current density (EQE–*J*) characteristics. The inset shows the normalized EQE at 1 mA/cm^2^ plotted as HTL and ETL thicknesses, and (**c**) current efficiency–luminance–power efficiency (CE–L–PE) characteristics.

Based on the determined EML thickness and EL spectrum, the optimal HTL (as TCTA) and ETL (as TmPyPB) thicknesses were derived through the optical path length calculation [13]. Based on the calculated optimal HTL and ETL thicknesses (HTL = 56 nm, ETL = 68 nm), their thicknesses were adjusted: HTL 56 to 80 nm and ETL 40 to 68 nm. As a result, similar to our previous report of ultra-thin EML green PHOLEDs, it was shown that the effect of improving the efficiency characteristics by electrical balance was more powerful than optical balance [13]. Figure 3 shows the EL characteristics of the devices optimized through HTL and ETL thickness control.

The reference devices (HTL 56 nm; ETL 54 nm) showed the characteristics of a driving voltage of 3.3 V at 1 cd/m^2^, a maximum EQE of 16.6%, a maximum current efficiency of 43.9 cd/A, and a maximum power efficiency of 32.7 lm/W. According to HTL and ETL thickness controls, the normalized EL efficiency characteristics are inserted as insets in Figure 3b. As the thickness of the HTL increased, the EQE at 1 mA/cm^2^ decreased. HTL 56 nm shows the highest EQE, and the EQEs of ETL 40 and 54 nm are almost identical. However, when the ETL thickness was increased to 80 nm, the efficiency decreased by about 15%. The devices of HTL 56 nm and ETL 54 nm were used as references because the EQE and EL characteristics were the best.

### 3.3. Ultra-Thin EML Blue PHOLEDs with a Light-Extraction Diffuser

**Figure 4:**
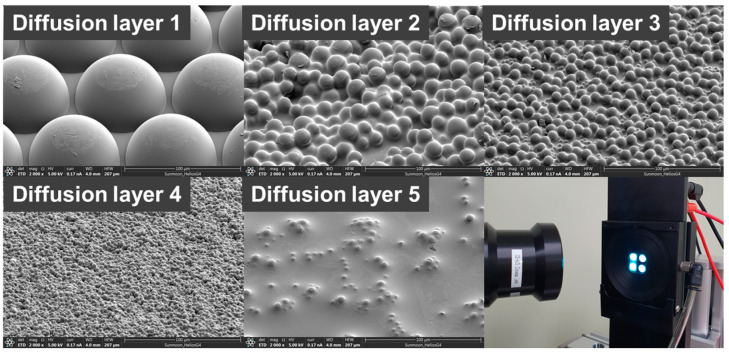
SEM images of diffusers and the real image of the blue PHOLED device.

The various diffusers were applied to improve the viewing angle and light-extraction characteristics. The applied diffusers had different thicknesses, transmittance values, pattern sizes, pattern shapes, packing types, and packing densities. The summarized characteristics of the diffusers are shown in Figure 4 and Table 1.

**Figure 5:**
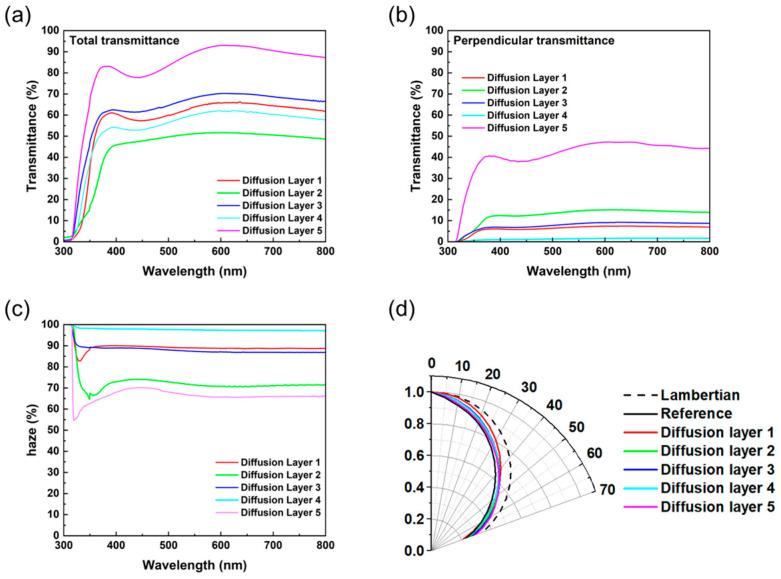
The optical characteristics of various diffusers: (**a**) total transmittance, (**b**) perpendicular transmittance, (**c**) haze, and (**d**) viewing angle.

As shown in Figure 4 and Table 1, each diffuser has a different pattern size, shape, packing type, and packing density. The pattern size of the diffusers is about 4 to 75 μm, and the pattern can be sorted by a periodically packed MLA and random arrangement, and further subdivided by shape into hemisphere and sphere. It can also be sorted by packing type and packing density. Diffuser 1 is a closed-packed type, diffusers 2 to 4 are randomly packed, and diffuser 5 is a random-embedded type. The packing density was high in all diffusers, except diffuser 5. To analyze the optical properties of the diffusers, the total transmittance (T_t_) and perpendicular transmittance (T_p_) were measured by UV-Vis and NIR spectrophotometers. The diffuse transmittance (T_d_) and haze were calculated using Equations (1) and (3). T_t_ and T_p_ at 470 nm are shown in Table 1, which is the peak of the emission wavelength of blue PHOLEDs. In Figure 5a–c, the T_t_, T_p_, and haze of each diffuser are presented. The T_t_ is expressed as the sum of T_p_ and the diffuse transmittance (T_d_), and the haze is expressed by the division of T_d_ by T_t_. The relevant formula is as follows:

(1) $$T_{t}=T_{p}+T_{d}$$

(2) $$T_{p}=\frac{Transmitted\;light}{Incident\;light}\;at\;incident\;angle=0{}^{\circ}$$

(3) $$Haze\left(\%\right)=\frac{T_{t}-T_{p}}{T_{t}}\times 100=\frac{T_{d}}{T_{t}}\times 100$$

The transmittance characteristics shown in Table 1 and Figure 5 have the same tendency in the visible-light wavelength region. Figure 5a,b show T_t_ and T_p_, respectively. T_d_ can be obtained through the relationship between T_t_ and T_p_. The haze characteristics are shown in Figure 5c. High haze means that T_d_ is high compared to T_t_, which means that more light is scattered laterally than the light that escapes in the forward direction [14]. To evaluate the diffuser-induced change in EL characteristics, the blue ultra-thin EML OLEDs were fabricated and utilized with diffusers. The devices were named in the same order of the diffusers, that is, OLEDs with diffusers 1–5 to devices 1–5. The fabricated devices showed different EL characteristics depending on the diffusers. Table 2 summarizes the EL efficiency and viewing angle improvement characteristics.

The device that showed the greatest improvement in the EQE was device 1, which was a diffuser with a 75 μm hemisphere MLA structure. Compared to the reference device, the EQE was improved by about 46.9% (* 1.47 fold), and the viewing angle was improved by about 5.1% compared to the reference device.

The previous reports of other groups showed that the hemisphere structure was advantageous for light-extraction purposes [15,16,17]. Device 1 with diffuser 1 having a closed packed/hemisphere structure showed a high efficiency level compared to the other diffusers with a random closed-packed or random embedded structure. Although diffuser 1 had a hexagonal closed-packed structure, it had a lower packing density than the random closed-packed spheres in diffusers 2–4. However, diffuser 1 showed the greatest improvement. Therefore, it is possible to conclude that the shape and size are more important than the packing density. In devices 2 to 4, the packing type of the diffusers was a random closed structure. In this packing type, it was confirmed that the efficiency improvement was increased as the size of the sphere was increased. However, compared to the reference device, the improvement of the viewing angle characteristics was not achieved. Finally, device 5, which was applied with diffuser 5, was a random embedded/sphere type, and all of the viewing angles and EQE improvements were insignificant. Through this, it was confirmed that the light-extraction characteristics differed depending on the packing type and packing density of the diffusers. On the other hand, it was confirmed that the change in CIE 1931 color coordinates for all devices compared to the reference device at 500 cd/m^2^ was at most 0.02, and it was confirmed that even if the diffusers were applied, the color coordinates were not significantly affected.

### 3.4. Investigation of the Correlation between Viewing Angle Profile and Light-Extraction Characteristics with Experimental and FDTD Simulations

**Figure 6:**
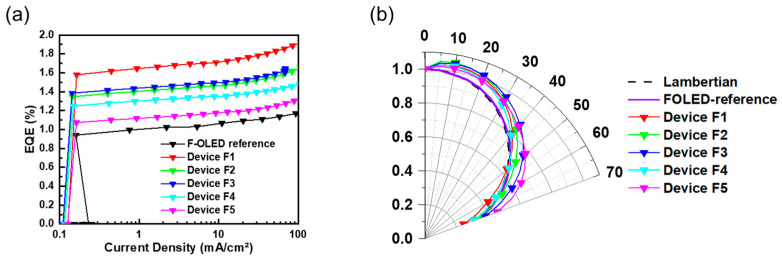
(**a**) Current density–external quantum efficiency (J–EQE) characteristics and (**b**) viewing angle characteristics of fluorescent OLEDs with various diffusers.

We confirmed that the viewing angle profile of blue PHOLEDs with ultra-thin EMLs was narrower than that of Lambertian light sources, as shown in Figure 5d. The viewing angle profile of the OLED device was determined by the optical factor of the OLED device (refractive index of materials; thickness) and the orientation of emitter molecules [18]. To analyze the efficiency enhancement characteristics of the diffuser according to the viewing angle profile in-depth, commonly used green, fluorescent OLEDs were fabricated, and various diffusers were applied. The fluorescent OLEDs were fabricated by the most well-known structure of ITO (185 nm)/NPB (60 nm)/Alq_3_ (60 nm)/LiF (1 nm)/Al (200 nm), and the fluorescent OLEDs with diffusers were named by order of device: F1–F5. Figure 6 shows the current density to EQE characteristics and viewing angle profiles of the green, fluorescent OLEDs with diffusers 1 to 5. Table 3 summarizes the EL efficiency and viewing angle improvement characteristics of the fluorescent OLEDs.

The efficiency improvement characteristics of the green, fluorescent OLEDs with diffusers showed a similar order to that of blue PHOLEDs with diffusers. Device F1 showed the greatest EQE enhancement by 1.61 times at 20 mA/cm^2^ compared to the FOLED reference device, and devices F2 to F5 showed 1.38-, 1.4-, 1.26-, and 1.01-fold EQE enhancements, respectively. The fluorescent OLEDs with diffusers showed greater EQE enhancements than PHOLEDs with diffusers. In particular, device F1 showed the greatest efficiency improvement compared with device 1, improving its light efficiency by about 13.6%. Diffuser 5 showed the greatest viewing angle improvement, and device F5 compared to device 5 showed an approximately 2.4-fold improvement.

The relatively small improvement in the EQE of the blue PHOLEDs with diffusers could be attributed to the difference in the viewing angle profiles between blue PHOLEDs and green, fluorescent OLEDs. In other words, it can be suggested that a viewing angle profile close to the Lambertian source or a wider viewing angle profile can achieve a greater EQE improvement.

**Figure 7:**
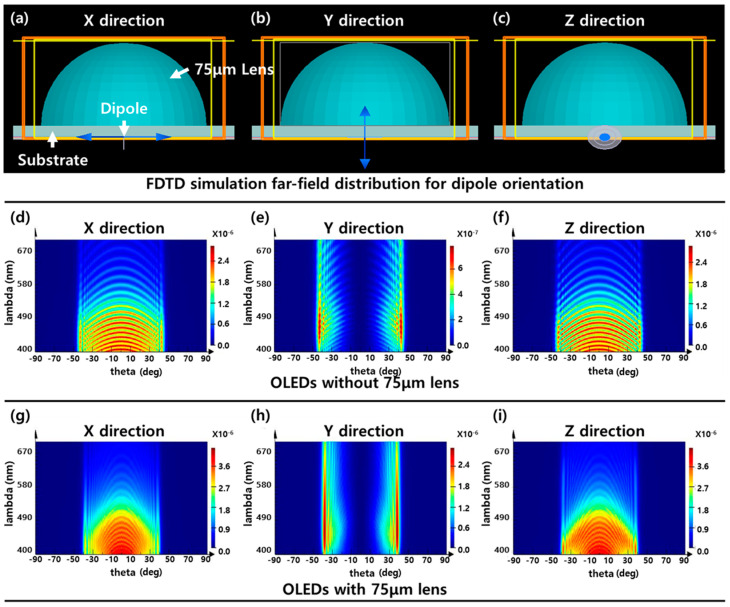
FDTD simulation model of fluorescent OLED structure with different dipole orientations in (**a**) X-, (**b**) Y-, and (**c**) Z-axis directions. The far-field distribution of OLEDs w/o a 75 μm lens based on dipole orientations in (**d**) X-, (**e**) Y-, (**f**) Z-axis directions and OLEDs w/a 75 μm lens based on dipole orientations in (**g**) X-, (**h**), Y-, (**i**) Z-axis directions.

For a theoretical verification, an FDTD simulation was conducted and analyzed for the light-extraction enhancement (LEE) characteristics according to the dipole distribution. Figure 7 shows the FDTD simulation results according to the presence or absence of the 75 μm lens and the dipole direction (Supplementary Materials Figure S2).

**Figure 8:**
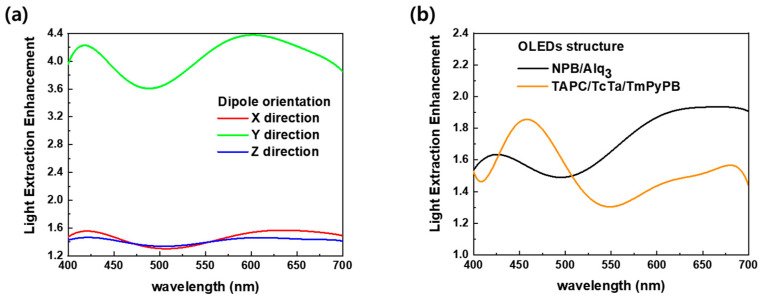
The light-extraction enhancement characteristics compared to wavelength, according to (**a**) dipole orientation and (**b**) OLED device structure.

An FDTD simulation was conducted based on the 75 μm hemispherical lens structure of diffuser 1 and the NPB/Alq_3_ structure, which is a fluorescent OLED. The substrate of the OLED device used in the FDTD simulation was set thinner than the actual substrate thickness to simplify the calculation, and one dipole was inserted. In general OLEDs, the light intensity is different depending on the orientation of the emitter molecules, and it is known that stronger emissions occur when the dipole is oriented horizontally to the substrate, rather than vertically [18]. Therefore, we compared the case where the dipole had directions of each of the X, Y, and Z axes through the FDTD simulation. The far-field distribution according to the angle and wavelength as the presence or absence of the 75 μm lens is plotted as a contour graph in Figure 7d–i. In the case of OLEDs without any light-extraction structures, as previously known, stronger light emission was shown in the dipole-aligned X- and Z-axis directions (horizontally) than the dipole in the Y-axis direction (vertically). The far-field intensity according to the horizontal and vertical orientations of the dipole differed by about an order of 1. In the OLEDs with lenses, there was no significant difference in the maximum emission intensity in the horizontal and vertical directions of the dipole; however, the forward emission was greater in the horizontal dipole orientation. However, the orientation of emitter molecules in actual OLEDs is difficult to be biased in a specific direction and is randomly arranged. Therefore, the light-extraction enhancement characteristics of dipole and random orientations are shown in Figure 8. In addition, simulations were conducted based on the TAPC/TCTA/TmPyPB structure to compare the LEE characteristics according to the difference in the device structure and refractive index or materials. Figure 8b shows the comparison of light-extraction enhancement between the NPB/Alq_3_ and TAPC/TCTA/TmPyPB structures.

As shown in Figure 8a, in the case of the dipole-oriented Y-axis direction, the highest maximum LEE of about 4.4 can be observed. However, as shown in the FDTD results in Figure 7, in the case of the dipole having a Y-axis orientation, which is vertical to the substrate, the far-field intensity is weaker than that of the horizontal orientation. That is, it was confirmed that the LEE characteristics could be considerably improved, even when the intensity of light emitted from the OLED was weak. Figure 8b shows that the light-extraction enhancement characteristics are different depending on the refractive index of the materials and OLED structure. The reason for the different LEE characteristics in the two structures is that the optical path determined by the refractive index inherent in the material and the thickness of the OLED device are different. That is since the optimal light-extraction wavelength band was determined according to the optical path, it was important to adopt a device structure and material suitable for the emission wavelength of the emitter.

In summary, The EL efficiency showed different dependence results on the shape and size of the structure and packing type and density, rather than the packing density. The EQE improvement was significantly affected by the shape and size of the structure, and the viewing angle was not affected by the packing density, structure size, or structure shape. So, it is possible to conclude that the selective and correlative improvements of the viewing angle and EL efficiency are possible by controlling the external diffuser. In addition, it was experimentally confirmed that EQE enhancement and viewing angle improvement characteristics could be selectively changed according to the unique viewing angle profile of OLEDs. As a result, the device using diffuser 1 showed an EQE of about 24.3%. The result of the reference device is high compared to the results of the blue PHOLEDs manufactured using common materials, and the maximum EQE of the device using MLA diffusers is very high in the published papers at present [19,20,21,22].

## 4. Conclusions

In this study, we fabricated ultra-thin EML blue PHOLEDs and applied various diffusers. A highly efficient blue ultra-thin EML PHOLED was investigated to achieve high EL efficiency. The fabricated ultra-thin blue OLEDs showed a driving voltage of 3.3 V at 1 cd/m^2^, a maximum EQE of 16.6%, a maximum current efficiency of 43.9 cd/A, and a maximum power efficiency of 32.7 lm/W. The diffusers with various thicknesses, transmittances, and morphological characteristics were applied to the reference device to improve the light-extraction and viewing angle characteristics. The characteristics of each diffuser and its device characteristics were investigated through systematic analyses. All the devices showed improvements in the viewing angle and efficiency. The EQE was improved 1.47-fold and the viewing angle was improved 1.06-fold, respectively. It was confirmed that EL efficiency improvement and viewing angle widening were selective. The devices with hemisphere MLA-based diffusers showed the greatest efficiency improvement and achieved a maximum EQE of 24.3%. This result means that the diffusers selectively contribute to improving the viewing angle characteristics and efficiency. Additionally, for an in-depth analysis, an FDTD simulation was performed for theoretical verification. As a result of the simulation, it was confirmed that the LEE characteristics were different depending on the orientation of the dipole and the device structure.

In summary, if the diffuser used for the light extraction of the optimal structure is applied through a systematic multi-aspects analysis, such as the packing type, packing density, and pattern size of the diffuser, this suggests that more highly efficient OLEDs can be created, and the efficiency and viewing angle are selectively improvable. Additionally, we suggest that a more highly efficient OLED can be created based on a proper device design, such as refractive index matching and device thickness.

## Figures and Tables

**Table 1:** Summarized characteristics of diffusers’ specifications.

	Diffusion Layer 1	Diffusion Layer 2	Diffusion Layer 3	Diffusion Layer 4	Diffusion Layer 5
Pattern size *	75 μm	~18 μm	~9 μm	~4 μm	~7 μm
shape	Hemisphere	Sphere			
Packing type	Closed-packed	Random closed-packed			Random embedded
Density	Medium	←	→	High	Low
T_t_	57.96%	48.53%	62.71%	54.19%	79.50%
T_p_	6.03%	12.75%	7.19%	1.19%	39.04%

* Diameter of sphere or hemisphere.

**Table 2:** Summary of blue PHOLED device characteristics with various diffusers applied: EQE, viewing angle improvement, and CIE 1931 color coordinate.

Device	EQE Enhancement *	Viewing Angle Improvement	CIE 1931 (x,y) @500 cd/m^2^ (Δreference)
Device 1	46.9% (* 1.47-fold)	5.1% (* 1.05-fold)	(0.151, 0.337) Δ(0.01, 0.01)
Device 2	35.5% (* 1.36-fold)	4.2% (* 1.04-fold)	(0.148, 0.336) Δ(0.00, 0.02)
Device 3	33.5% (* 1.34-fold)	5.6% (* 1.06-fold)	(0.148, 0.338) Δ(0.00, 0.01)
Device 4	20.4% (* 1.2-fold)	3.6% (* 1.04-fold)	(0.151, 0.336) Δ(0.01, 0.01)
Device 5	1.8% (* 1.02-fold)	4.8% (* 1.05-fold)	(0.146, 0.347) Δ(0.00, 0.00)

* The EQE enhancement was obtained by comparing the maximum EQE of each device and the reference device.

**Table 3:** Summary of green, fluorescent OLED device characteristics with various diffusers applied: EQE and viewing angle improvement.

Device	EQE @ 20 mA/cm^2^	EQE Enhancement *	Viewing Angle Improvement
Reference	1.09	0% (* 1.00-fold)	0% (* 1.00-fold)
Device F1	1.75	60.50% (* 1.61-fold)	2.08% (* 1.02-fold)
Device F2	1.50	37.60% (* 1.38-fold)	7.38% (* 1.07-fold)
Device F3	1.53	40.30% (* 1.40-fold)	11.16% (* 1.11-fold)
Device F4	1.39	25.60% (* 1.26-fold)	3.00% (* 1.03-fold)
Device F5	1.19	0.90% (* 1.01-fold)	11.36% (* 1.11-fold)

* EQE enhancement was obtained by comparing the maximum EQE of each device and the reference device.

## Data Availability

Not applicable.

## Supplementary Materials

The following supporting information can be downloaded at: https://www.mdpi.com/article/10.3390/nano13162357/s1, Figure S1. Transmittance measurement mechanism. Figure S2. FDTD Simulation structure.
