# Supplementary material for: Highly Efficient Ultra-Thin EML Blue PHOLEDs with an External Light-Extraction Diffuser
Source: Nanomaterials (Basel). 2023 Aug 17;13(16):2357. doi: 10.3390/nano13162357 (PMC10458805; doi:10.3390/nano13162357)
Supplement: Supplementary file 1 [file nanomaterials-13-02357-s001.zip › nanomaterials-2523271-supplementary.pdf]

Supplementary Material

Highly efficient ultra-thin EML blue PHOLEDs with the external light extraction diffuser

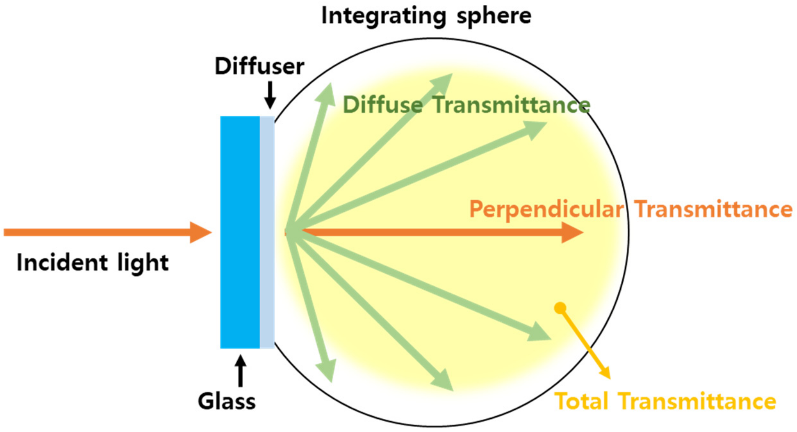

Figure S1. Transmittance measurement mechanism

Perpendicular transmittance, diffuse transmittance, and total transmittance were analyzed in the visible-light wavelength range of 300 to 800 nm using a UV-Vis NIR Spectrophotometer (Cary 5000, Agilent Technologies, Inc.). At this time, we used an integrating sphere to derive the diffuse transmittance value as a value to analyze the light scattering effect of the diffuser. The integrating sphere is mainly used to analyze diffuse transmittance, and measures the incident light entering the sphere (ie, the degree to which light is scattered after passing through the sample). Using this equipment, the values of perpendicular transmittance and total tranmsittance can be obtained, and the diffuse transmittance and haze are calculated by equations (1) and (2), respectively.

$$Diffuse\ Tranmsittance = Total\ Transmittance - Perpendicular\ Transmittance \quad \text{– equation (1)}$$

$$Haze\ (\%) = \frac{Diffuse\ Transmittance}{Total\ Transmittance} * 100\% \quad \text{- equation (2)}$$

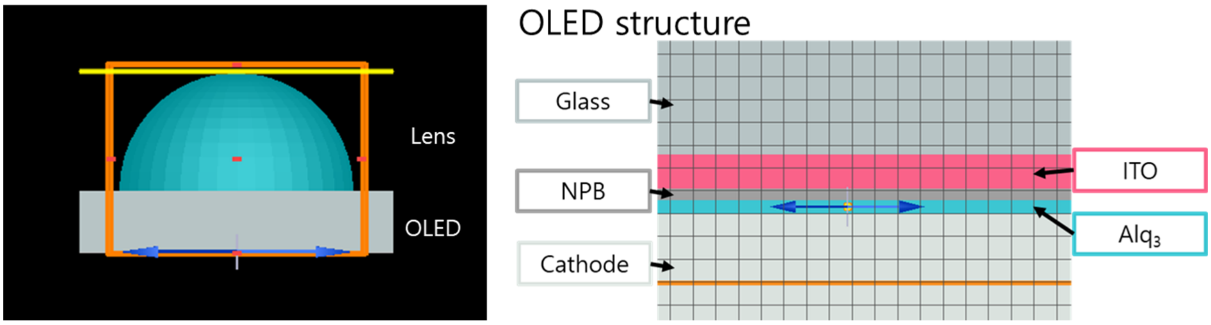

Figure S2. FDTD Simulation structure

The FDTD simulation was performed with a 2-dimensional fluorescent OLEDs with a structure of diffuser 1/Glass/ITO (185 nm)/NPB (60 nm)/Alq_3 (60 nm)/Cathode (200 nm). The material data of each layer used the *n* and *k* values measured according to the wavelength using a thin-film measurement system (F20, Filmetrics, Inc.). In order to match the optical properties with the actual device, the boundary condition was set to PML designed to absorb incident light with minimal reflections for the edge of the structure and metal for the end of the cathode. The device was simulated each of the x, y, and z axis (dipole orientation) using a dipole source to consider the Lambertian emission characteristics of OLEDs, and the light extraction efficiency obtained the LEE ratio for the two devices with and without patterns.
